# Chromatin profiling and state predictions reveal insights into epigenetic regulation during early porcine development

**DOI:** 10.1186/s13072-024-00542-w

**Published:** 2024-05-21

**Authors:** Sarah M. Innis, Ryan A. Cabot

**Affiliations:** Department of Animal Sciences, Purdue University, West Lafayette, IN 47907 USA

**Keywords:** Epigenetics, Histone modifications, Chromatin remodeling, CUT&RUN, Development

## Abstract

**Background:**

Given their physiological similarities to humans, pigs are increasingly used as model organisms in human-oriented biomedical studies. Additionally, their value to animal agriculture across the globe has led to the development of numerous studies to investigate how to improve livestock welfare and production efficiency. As such, pigs are uniquely poised as compelling models that can yield findings with potential implications in both human and animal contexts. Despite this, many gaps remain in our knowledge about the foundational mechanisms that govern gene expression in swine across different developmental stages, particularly in early development. To address some of these gaps, we profiled the histone marks H3K4me3, H3K27ac, and H3K27me3 and the SWI/SNF central ATPase BRG1 in two porcine cell lines representing discrete early developmental time points and used the resulting information to construct predicted chromatin state maps for these cells. We combined this approach with analysis of publicly available RNA-seq data to examine the relationship between epigenetic status and gene expression in these cell types.

**Results:**

In porcine fetal fibroblast (PFF) and trophectoderm cells (PTr2), we saw expected patterns of enrichment for each of the profiled epigenetic features relative to specific genomic regions. H3K4me3 was primarily enriched at and around global gene promoters, H3K27ac was enriched in promoter and intergenic regions, H3K27me3 had broad stretches of enrichment across the genome and narrower enrichment patterns in and around the promoter regions of some genes, and BRG1 primarily had detectable enrichment at and around promoter regions and in intergenic stretches, with many instances of H3K27ac co-enrichment. We used this information to perform genome-wide chromatin state predictions for 10 different states using ChromHMM. Using the predicted chromatin state maps, we identified a subset of genomic regions marked by broad H3K4me3 enrichment, and annotation of these regions revealed that they were highly associated with essential developmental processes and consisted largely of expressed genes. We then compared the identities of the genes marked by these regions to genes identified as cell-type-specific using transcriptome data and saw that a subset of broad H3K4me3-marked genes was also specifically expressed in either PFF or PTr2 cells.

**Conclusions:**

These findings enhance our understanding of the epigenetic landscape present in early swine development and provide insight into how variabilities in chromatin state are linked to cell identity. Furthermore, this data captures foundational epigenetic details in two valuable porcine cell lines and contributes to the growing body of knowledge surrounding the epigenetic landscape in this species.

**Supplementary Information:**

The online version contains supplementary material available at 10.1186/s13072-024-00542-w.

## Background

Epigenetics involves the study of the genome-wide presence of heritable gene expression modifiers capable of influencing transcription without changing the existing DNA sequence. Histone modifications such as methylation and acetylation represent a significant group of epigenetic features within the epigenome, and together with chromatin remodeling complexes, they contribute to the precise control of chromatin architecture and, by extension, the accessibility of genes to transcriptional machinery. Studying how the epigenome regulates gene expression during the earliest stages of organism development can reveal findings with broad implications for developmental biology, disease research, and regenerative medicine.

As a model organism, the pig is conveniently poised to contribute insights into both human health and animal agriculture. Pigs are an increasingly valuable biomedical model due to their similarities in mature organ size, metabolic physiology, and immune function to humans, especially as xenotransplantation of pig-derived tissues grows in prevalence of practice [1, 2]. In addition, several transgenic pig lines have been established to facilitate the study of several human diseases, including cystic fibrosis, diabetes mellitus, and various neurodegenerative and cardiovascular conditions [3–9], many of which were established via somatic cell nuclear transfer (SCNT) using cultured porcine fetal fibroblasts (PFFs) as nuclear donors. As a food animal, pigs remain a staple livestock species and food source worldwide, and recent research has endeavored to explore the creation of pigs that are faster growing, more feed-efficient, and less susceptible to stress and disease [10–12]. This includes studying the roles of the uterus and placenta in conferring nutrition *in utero* and providing an environment conducive to embryonic and fetal growth. Indeed, several studies focusing on increasing our understanding of placental physiology and gestational pathologies have been performed using the pig as a model organism, many of which have utilized a line of porcine trophectoderm (PTr2) cells for in vitro experiments [13–17].

Despite the promise of their utility as a biomedical model and widely-established value in food animal agriculture, many knowledge gaps remain concerning the porcine epigenome, particularly across different developmental time points. While a significant portion of porcine epigenome information has been obtained using information from DNA methylation studies, some efforts have been made to develop chromatin landscape maps across different swine tissues [18–21], especially as part of the broader Functional Annotation of Animal Genomes (FAANG) initiative [22]. However, to date, few studies have evaluated the epigenetic landscape or provided functional annotation details in porcine cells from early developmental time points. To address this dearth of information and contribute to growing efforts to generate epigenetic annotation data in swine models, we used CUT&RUN followed by next-generation sequencing (NGS) to evaluate the state of the histone modifications H3K4me3, H3K27ac, H3K27me3, and the evolutionarily- conserved SWI/SNF chromatin remodeling complex central ATPase BRG1 (also known as SMARCA4) in PTr2 and PFF cells. Using this data, we constructed genome-wide predicted chromatin state map for these cell lines. From these findings, we identified regions of the genome marked with broad H3K4me3 signal and annotated them to examine their potential roles in governing cell function and identity. Taken together, the present research provides an in vitro basis for obtaining epigenetic information in two discrete early developmental time points in swine and enhances our understanding of how specific histone modification patterns may guide cell identity and function.

## Methods

Unless otherwise stated, all chemicals were procured from Sigma Chemical Company, St. Louis, MO, USA. All cell culture reagents were obtained from Thermo Fisher Scientific, Waltham, MA, USA. Furthermore, with the exception of some antibodies, all CUT&RUN reaction reagents were purchased from EpiCypher, Durham, NC, USA.

### Cell lines and culture conditions

PFFs were harvested from a porcine conceptus on day 40 of gestation as previously described [23]. Cells were cultured in DMEM supplemented with 15% FBS, 1% L-glutamine, 1% sodium pyruvate, 1% MEM-nonessential amino acids, and 1% penicillin-streptomycin and then collected at passage 5 for use in this study. Non-primary PTr2 cells were grown as previously described [24] in phenol red-free DMEM-F12 supplemented with 5% FBS, 0.1 units/mL bovine insulin, 1% L-glutamine, and 1% penicillin-streptomycin. Both cell lines were cultured in 150 cm^2^ cell culture flasks at 39°C, 5% CO_2_, and 100% humidity. Cells were passaged at 90% confluency using trypsin/EDTA and were at passage 60 at the time of use in these experiments.

### Antibodies

The following antibodies were used in this study: CUTANA Rabbit IgG (13–0042, EpiCypher, Durham, NC), Anti-Histone H3 trimethyl K4 (13–0041, EpiCypher), Anti-BRG1/SMARCA4 (13-2002, EpiCypher), Anti-Histone H3 acetyl K27 (ab4729, Abcam, Cambridge, United Kingdom), Anti-Histone H3 trimethyl K27 (07-449, Sigma).

### CUT&RUN

The CUT&RUN assay was performed as previously described [25]. To elaborate, cells were harvested using trypsin/EDTA, counted on a hemacytometer, and then aliquoted at 5 × 10^5^ cells per sample onto magnetic Concanavalin A beads (21-1401). For each sample of immobilized cells, 0.5 µg of primary antibody was added, and the samples were incubated overnight on an orbital rotator (Boekel Scientific, Philadelphia, PA, USA) at 4 °C. Following incubation, bead-bound cells were permeabilized, and 2.5 µL of protein A/G micrococcal nuclease (pAG-MNase) (15-1016) was added per sample and incubated for 10 min at room temperature (RT). The pAG-MNase was activated by the addition of 1 µL 100 nM CaCl_2_ (21-1007), and samples were incubated on an orbital rotator for 2 h at 4 °C to allow the chromatin cleavage reaction to proceed. At the end of the 2-hour incubation period, pAG-MNase activity was terminated by the addition of Stop Buffer (21-1003) containing *E. coli* Spike-in DNA (18-1401) and a 10-minute incubation period at 37 °C. After this incubation period, CUT&RUN enriched DNA was purified using DNA Binding Buffer (21-1008), DNA Wash Buffer (21-1009), and DNA Elution Buffer (21-1010) provided in the CUT&RUN kit. The purified CUT&RUN DNA was then quantified on a Qubit fluorometer (ThermoFisher) before proceeding to library preparation. Libraries were prepared using the NEBNext Ultra II DNA Library Prep Kit for Illumina (E7645S, New England Biolabs (NEB), Ipswich, MA, USA), NEBNext Multiplex Oligos for Illumina (Sets 1–4) (Set 1: E7335S, Set 2: E7500S, Set 3: E7710S, Set 4: E7730S, NEB), and Agencourt AMPure XP Beads (A63880, Beckman Coulter, Brea, CA). Libraries were quantified on a Qubit fluorometer and were evaluated for size on a TapeStation system (Agilent Technologies, Santa Clara, CA, USA). Paired-end sequencing (150 bp read length) was performed on an Illumina NovaSeq 6000 system (Illumina, San Diego, CA, USA) at a read depth of 5–8 million reads per library.

### CUT&RUN sequencing data analysis

Adapter sequences were removed from demultiplexed, paired-end Illumina reads (as FASTQ files) using TrimGalore (v. 0.6.7) [26] with the - -fastqc parameter. Trimmed reads were aligned to the pig genome assembly Sscrofa11.1 (GenBank accession GCA-000003025.6) and the *E. coli* K12, MG1655 reference genome (obtained from https://support.illumina.com/sequencing/sequencing_software/igenome.html) using Bowtie2 (v. 2.4.5) [27] and the options --local, --very-sensitive, --no-unal, --no-mixed, and --no-discordant. The resulting SAM files (aligned to Sscrofa11.1) were converted to BAM files, sorted by genomic coordinates, and indexed using SAMtools (v. 1.15.1) [28] sort and index, respectively. BAM files were filtered to remove unmapped reads, improperly paired reads, and reads with a MAPQ score < 20 using SAMtools view. Using the *E. coli* alignment files, normalization factors were calculated as previously described [29]. Normalized bigWig files were created using deepTools (v. 3.5.1) [30] bamCoverage with the --scaleFactor option set to the corresponding normalization factor calculated previously, a --binSize of 20, and a --smoothLength of 60. Additionally, reads were extended and centered. Coverage tracks were visualized using the Broad Institute’s Integrated Genomics Viewer (IGV) [31]. A BED file containing the gene coordinates for the Sscrofa11.1 assembly was obtained using the UCSC table browser [32]. Reference point matrices were generated using deepTools computeMatrix and profile plot and heatmap visualizations of these matrices were generated using deeptools plotProfile and plotHeatmap, respectively. MACS2 was used to call peaks with - - format BAMPE and - - keep-dup all, and corresponding IgG BAM files were included as controls [33]. The - - broad parameter was included for H3K27me3 samples. Peak detection was passed on a minimum FDR cutoff (q-value) of 0.05. Bedtools (v. 2.30.0) intersect was used to compare replicate and sample peaks. Replicate PCA plots and correlation heatmaps were created using the R Bioconductor package DiffBind (v. 3.8.4) using read count data normalized by library size [34, 35]. Binding site overlaps between replicates and samples were also evaluated using DiffBind. Binding site annotation was performed using the Bioconductor annotation packages ChIPseeker (v. 1.34.1) [36] and ChIPpeakAnno (v. 3.32.0) [37]. For Gene Ontology overrepresentation testing, one-sided Fisher’s exact test was used to test for statistical significance (*p* ≤ 0.05), and Benjamini‒Hochberg correction (FDR) was performed to obtain adjusted *p*-values (q ≤ 0.05). For chromatin state predictions, BAM files were converted to BED with Bedtools bamtobed and binarized using BinarizeBed from ChromHMM (v. 1.25) [38]. Models were generated using the ChromHMM LearnModel command, and output BED files were visualized in IGV. Motif enrichment analysis and determination of enriched motif *p*-values using cumulative binomial distribution were performed using findMotifsGenome.pl in HOMER with default parameters.

### RNA-seq data analysis

RNA-seq data were obtained from the NIH BioProject database. Raw FASTQ files were sourced from accessions PRJNA798047 and PRJNA778857 and then aligned to the Sscrofa11.1 reference genome (NCBI RefSeq assembly GCF_000003025.6). Sscrofa11.1 was indexed with the hisat2-build command, and read alignments were performed using HISAT2 (v. 2.2.1) [39, 59]. Counts were obtained with HTSeq-count [40, 60] using the NCBI Sscrofa11.1 annotation features (GTF) genome file and with the mode option set to intersection-nonempty. Limma [41, 61] was used to obtain normalized read count data for each count file using a *P* value adjusted threshold of 0.05 (Benjamini‒Hochberg correction) and trimmed mean of M values (TMM) normalization [42]. Tissue-specific genes were identified as previously described [43, 44]. Briefly, a biological category (i.e. fibroblast) was assigned to each sample according to material source for each species. A *t-*statistic was calculated for each gene by excluding expression data belonging to other cells in the same biological category, then each gene list was ranked by *t*-statistic. The top 10% of genes as ranked by *t*-statistic were defined as likely tissue-specific genes and used for downstream comparisons between cell types.

### RT-qPCR characterization of PTr2 cells

RNA was extracted from PFF (passage 5) and PTr2 (passage 60) cells with the Direct-zol RNA Miniprep kit (Zymo Research, Irvine, CA, USA). Total RNA was quantified on a Nanodrop ND-1000 (ThermoFisher) device, and integrity was evaluated via denaturing gel. RNA was reverse-transcribed with the High-Capacity cDNA Reverse Transcription Kit (Applied Biosystems, Foster City, CA, USA). Primers targeting genes of interest were designed with the NCBI Primer-BLAST (Table 1) and were produced by IDT (Integrated DNA Technologies, Coralville, IA, USA). All primer validation and qPCR runs were performed on a CFX Connect Real-Time System (Bio-Rad, Hercules, CA, USA). Primer efficiency for each target was between 95 and 105%, with no evidence of multiple amplicons seen in the melt curve analysis. For relative quantification of gene expression, qPCR was performed in technical triplicate for each cell type with the SsoAdvanced Universal SYBR Green SuperMix (Bio-Rad). Transcript data was normalized to GAPDH, and PFF cells were used as the calibrator. The 2^−ΔΔCt^ method was used to obtain relative log fold change expression values for PTr2 cells.

**Table 1:** Primer sequences for genes of interest used for qPCR validation of PTr2 cells

Target Gene	RefSeq Gene ID	Forward Primer	Reverse Primer	Annealing Temperature (°C)	Amplicon Size (bp)
FGFR2/KGFR	396762	5’-AAGATGATGCCACAGAGAAAGA-3’	5’-CAGGCTCCGAGGAGATTTATG-3’	62	103
GAPDH	110260753	5’-GGTGAAGGTCGGAGTGAACG-3’	5’-TGACTGTGCCGTGGAATTTG-3’	62	101
KRT7	100626722	5’-ACCAGACCAAGTTTGAGACC-3’	5’-TCGGTTCATCTCCGCAATC-3’	62	103
KRT8	100152077	5’-GGTTCTGGAGACCAAATGGAA-3’	5’-CGCCGGAGGTTGTTGATATAG-3’	62	96
ITGA4	100521477	5’-GGTGGCTGGAGAATGAGAAA-3’	5’-ACTGGTACACACCAAGTTAAGG-3’	62	103
ITGA5	100155091	5’-TCCTACATTACCAGAGCAAGAG-3’	5’-CAGGTCTGGCACACAGATATT-3’	62	90
ITGB1	397019	5’-ACCTTATGGACCTCTCCTACTC-3’	5’-ACTCTGAAGTAATCCTCCTCATTTC-3’	62	99

## Results

### Global enrichment patterns of H3K4me3, H3K27ac, H3K27me3, and BRG1 in PFF cells

**Fig. 1:**
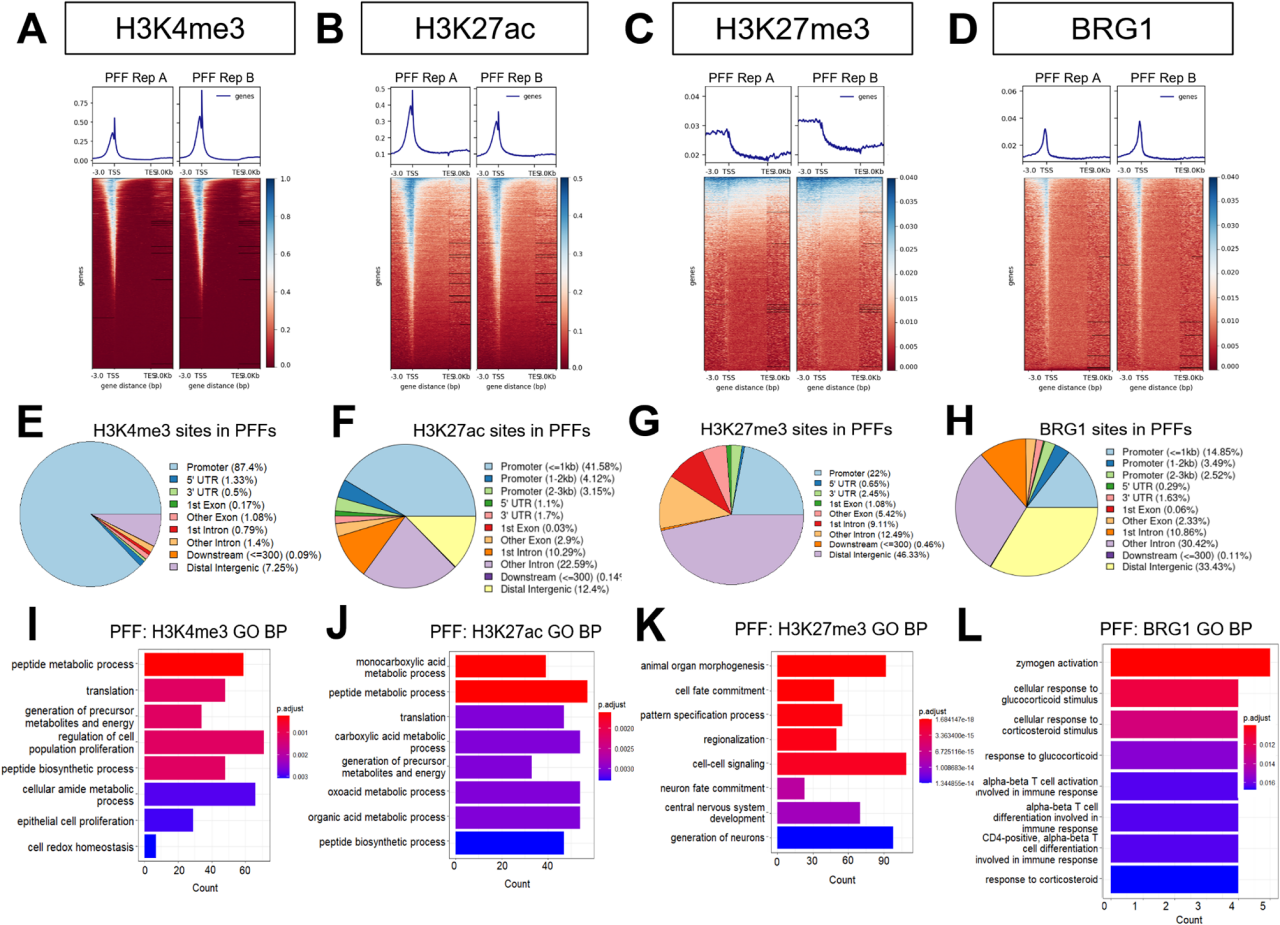
Genomic localization and functional annotation of epigenetic features in PFF cells. Enrichment of (**A**) H3K4me3, (**B**) H3K27ac, (**C**) H3K27me3, and (**D**) BRG1 relative to global TSS and TES locations. Genomic feature annotation of global (**E**) H3K4me3, (**F**) H3K27ac, (**G**) H3K27me3, and (**H**) BRG1 binding. GO biological process overrepresentation analysis (*P* < 0.05 using one-sided Fisher’s exact test; adjusted *P* < 0.05 after Benjamin-Hochberg FDR) for (**I**) H3K4me3, (**J**) H3K27ac, (**K**) H3K27me3, and (**L**) BRG1

H3K4me3 was strongly enriched around transcriptional start sites (TSSs) and had minimal detectable enrichment outside of these regions (Fig. 1A). The majority of H3K27ac signal was also present around global TSSs, but a larger proportion of H3K27ac binding was apparent outside TSS and gene bodies relative to H3K4me3 (Fig. 1B). Some H3K27me3 signal was found proximal to TSS regions, but enrichment was also broadly present outside of known gene coordinates (Fig. 1C). BRG1 signal showed a similar enrichment pattern to that of H3K27ac, albeit with lower overall signal abundance (Fig. 1D). Overall, these observations were supported by genomic region annotation of the selected epigenetic features, and the largest shares of each annotation category are included here. Nearly 90% (87.4%) of H3K4me3 signal was in gene promoter regions (Fig. 1E), consistent with the expected enrichment characteristics for this mark. Of the regions identified as represented in the H3K27ac annotation, the largest share of H3K27ac signal was present in promoter regions (41.58%), though just under a third (32.88%) of H3K27ac sites were in introns, and 12.40% were in distal intergenic regions (Fig. 1F). Approximately 22% of H3K27me3 signal was detected in promoter regions, while 46.33% of signal was in distal intergenic regions (Fig. 1G). For BRG1, 14.85% of signal was in promoter regions, while 41.28% was in introns, and around a third (33.43%) was in distal intergenic regions. Gene ontology (GO) analysis of each of these features showed that their enrichment was associated with a broad range of biological processes, including multiple terms concerned with key aspects of developmental progression (Fig. 1I-L). Furthermore, comparison of the epigenetic data to publicly-available RNA-seq data showed that the genes identified in the top 3 GO biological processes for H3K4me3 and H3K27ac were largely expressed and had higher expression levels relative to genes enriched for H3K27me3 (Supplementary Fig. S1A-C).

### Characterization of PTr2 cells

As the PTr2 cells used in this work were at a later passage at the time of CUT&RUN, we first wanted to characterize these cells using select marker genes. Both the cytokeratin genes *KRT7* and *KRT8* were included in this characterization, as they have both been shown to be present in PTr2 cells [45, 46]. Additionally, the gene keratinocyte growth factor receptor (*KGFR*) gene, also known as FGFR2, for fibroblast growth factor receptor 2 (*FGFR2)* was included, as this gene is known to be expressed in PTr2s [46, 47]. Several integrins have also been shown to be expressed in PTr2 cells, such as *ITGA5* [48, 49] and *ITGB1* [49]. It has been reported, however, that the integrin *ITGA4* is not expressed in this cell line [49], so this gene target was also included to further validate the character of these PTr2 cells. Exploration of publicly-available RNA-seq data for this cell line confirmed that *KRT7* and *KRT8* were both highly expressed in this cell line, as was *ITGB1* (Supplementary Fig. S2A). *FGFR2* also appeared to be expressed, albeit at a lower level than the keratin genes. *ITGA5* expression data suggested that this gene is likely lowly expressed in PTr2s, while *ITGA4* is not expressed (Supplementary Fig. S2A). *RT*-qPCR data confirmed that both *KRT7* and *KRT8* were highly expressed in the PTr2 cells, consistent with what would be expected for trophectoderm cells given their epithelial character (Supplementary Fig. S2B). Analysis of transcriptional data for *KGFR/FGFR2* indicated that this gene was more moderately expressed in PTr2 cells than *KRT7* and *KRT8* and that expression levels were similar between PTr2 and PFF cells. For the integrins, *ITGA5* appeared to have a considerably lower level of expression in PTr2 cells relative to PFFs. Previous research has shown that while *ITGA5* expression is detectable in PTr2 cells, it is comparatively lower than that of other integrins such as *ITGB1* [49]. Indeed, while PTr2 and PFF cells had a similar level of *ITGB1* expression, examination of the amplification data for this gene indicated that it was more highly expressed than *ITGA5* in PTr2s (Supplementary Fig. S2B). *ITGA4* expression was very low in PTr2 cells, and this observation is consistent with what has been reported in the literature [49]. Taken together, these results indicate that the PTr2 cells used in this study have retained PTr2 character despite their passage status.

### Global enrichment patterns of H3K4me3, H3K27ac, H3K27me3, and BRG1 in PTr2 cells

**Fig. 2:**
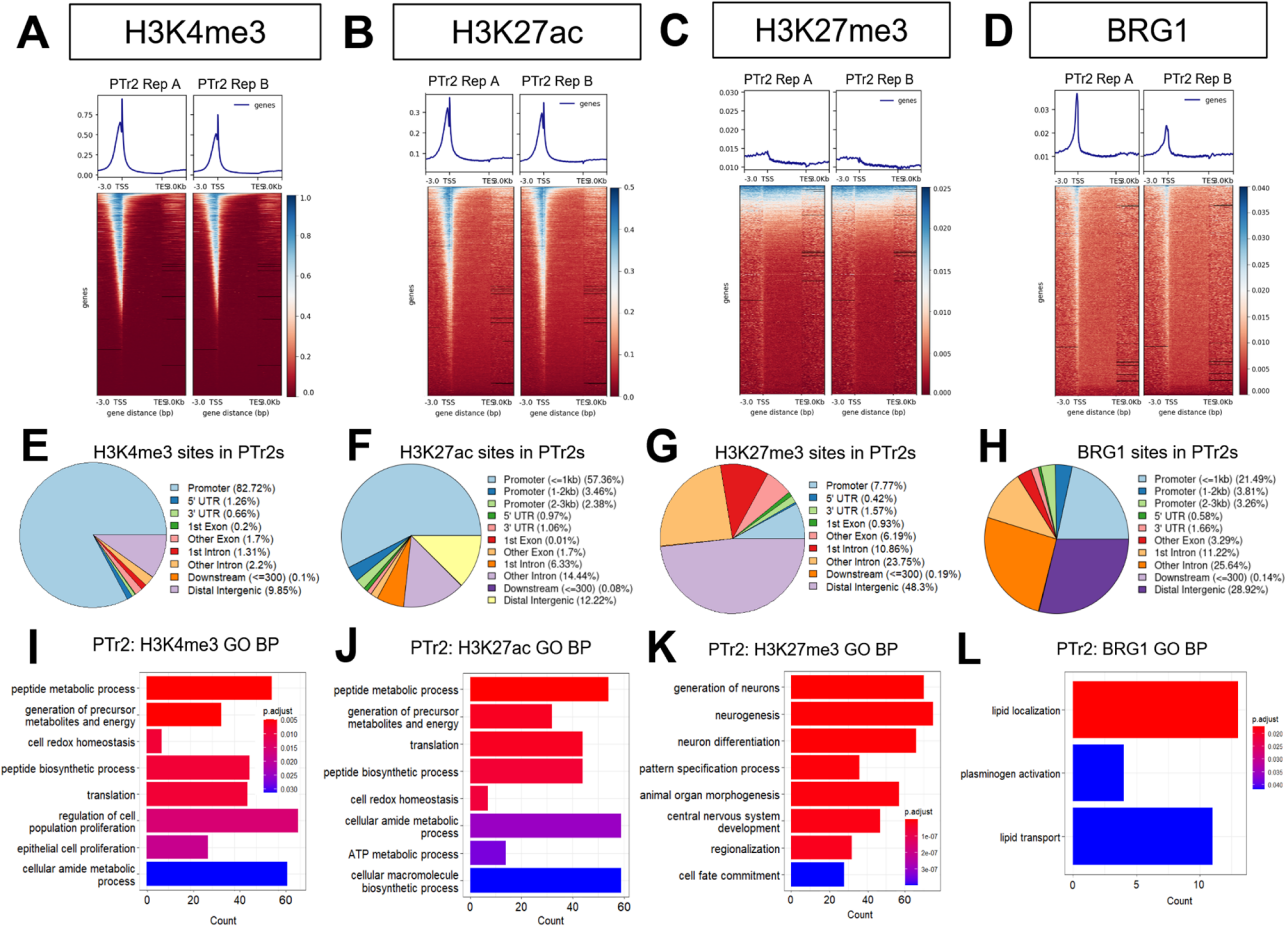
Genomic localization and functional annotation of epigenetic features in PTr2 cells. Enrichment of (**A**) H3K4me3, (**B**) H3K27ac, (**C**) H3K27me3, and (**D**) BRG1 relative to global TSS and TES locations. Genomic feature annotation of global (**E**) H3K4me3, (**F**) H3K27ac, (**G**) H3K27me3, and (**H**) BRG1 binding. GO biological process overrepresentation analysis (*p* < 0.05 using one-sided Fisher’s exact test; adjusted *P* < 0.05 after Benjamini-Hochberg FDR) for (**I**) H3K4me3, (**J**) H3K27ac, (**K**) H3K27me3, and (**L**) BRG1

Enrichment patterns for the selected epigenetic features were broadly similar between PFF and PTr2 cells. H3K4me3 signal was again strongly enriched around global TSS locations (Fig. 2A). H3K27ac signal was clearly detected at and immediately around TSSs, and the presence of this mark outside of gene coordinates was also apparent (Fig. 2B). As with PFF cells, H3K27me3 signal showed a small spike in enrichment around TSSs, but overall, signal for this mark seemed to be primarily outside of TSS and gene bodies (Fig. 2C). Additionally, PTr2 BRG1 signal also showed clear enrichment around TSSs, though heatmap patterns suggested that a majority of BRG1 signal was likely present outside of these sites (Fig. 2D). Genomic region annotation of H3K4me3 showed comparable results to what was seen in PFFs cells, with 82.72% of enrichment sites for this mark detected in promoter regions. A larger share of H3K27ac signal was detected in PTr2 cell promoters (57.36%) than what was observed in PFF cells, though less disparity was seen in the proportion of signal split between introns and distal intergenic regions PTr2s, with 20.77% and 12.22% of H3K27ac signal detected in these regions, respectively (Fig. 2F). H3K27me3 signal in PTr2 promoter regions was lower than that of PFFs, at 7.77%, while the proportion of signal detected in distal intergenic regions was highly similar, at 48.3% (Fig. 2G). For BRG1 in PTr2 cells, 21.49% of BRG1 signal was detected at global promoter regions, while 36.86% of signal was found in introns and 28.92% in distal intergenic regions (Fig. 2H). Once again, functional annotation of the global enrichment for these epigenetic features in PTr2 cells showed that a variety of different biological processes were enriched in the feature data, such as metabolic processes and control of cell proliferation (Fig. 2I-L). Similar to what was seen in PFF cells, the RNA-seq expression values for genes in the top 3 biological process categories for each histone mark showed that the vast majority of genes marked by H3K4me3 and H3K27ac were expressed, while the converse was seen for genes marked with H3K27me3 (Supplementary Fig. S3A-C).

### Genome-wide chromatin state predictions in PFF and PTr2 cells

**Fig. 3:**
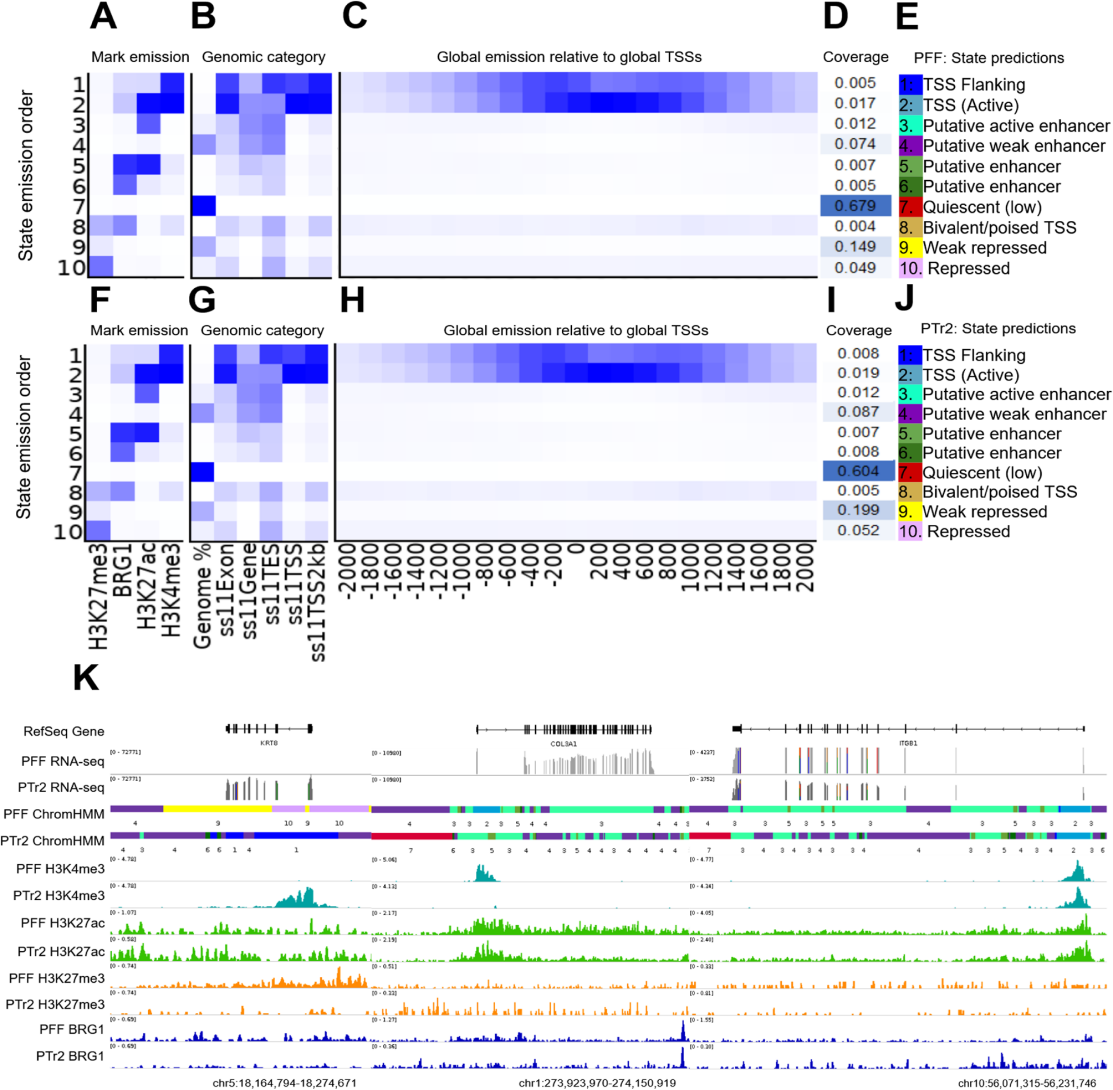
Chromatin state prediction for PFF (**A-E**) and PTr2 (**F-J**) cells, (**A,F**) Emission probabilities for each profiled feature across 10 states. Darker blue indicates a higher emission probability (0–1 ), (**B, G**) Genomic coverage of each state relative to the defined genomic features. (**C, H**) Coverage of each state relative to global TSS coordinates. (**D,I**) Genomic coverage of each emission state relative to other states. (**E,J**) Predictions of chromatin state identities. (**K**) Genome browser view of PFF and PTr2 predicted chromatin states and associated epigenetic feature enrichment at select loci. Note that the color and numeric code in the predicted state tracks corresponds to the colors and numbers assigned in panels **E** and **J**

Using the alignment files for H3K4me3, H3K27ac, H3K27me3, and BRG1, we constructed a chromatin state prediction model for each cell type. A total of 10 different states were defined using a combination of the previously discussed enrichment details and combined signal annotation predictions for each feature to generate a genome-wide view of putative chromatin states in PFFs and PTr2s. The 10 distinct chromatin states defined here represented promoter regions (TSS Flanking, TSS Active, and Bivalent/poised TSS), putative enhancers (including predicted enhancer strength for Putative active and Putative weak), quiescent/lowly-transcribed regions (Quiescent(low)), and repressed genomic regions (Repressed, Weak repressed). Overall, while PFF and PTr2 cells were analyzed separately, the chromatin state predictions showed similar annotation patterns between the two cell lines (Fig. 2A-J; Supplementary Fig. S4A-F). As such, the order of predicted chromatin states was the same between these cells (Fig. 2E, J). The chromatin state predictions were in agreement with genome browser views of epigenetic feature enrichment and the accompanying RNA-seq data, including at select loci representing genes with known expression in both (*ITGB1*; confirmed by RT-qPCR*)* cell lines or only one cell line, as in the case of *KRT8* in PTr2 cells (confirmed by RT-qPCR) and *COL3A1* in the fibroblasts [50](Zoppi et al., 2004; Li et al. 2021) [51] (Fig. 3K).

To further explore how the chromatin state prediction approach could be leveraged to reveal insights into the gene expression regulatory landscape, we obtained the genomic coordinates for predicted enhancer regions in both cell lines and conducted a motif enrichment analysis to investigate transcription factor binding at these regions. PFF and PTr2 cells shared a common overrepresented motif in FOSL1::JUND (AP-1), and both cell lines had a TEAD motif, albeit corresponding to different members of the TEAD family. The remaining motifs showed no overlap in identity between the cell types.

### Broad regions of H3K4me3 signal at predicted TSS regions mark essential development and tissue-specific genes

**Fig. 4:**
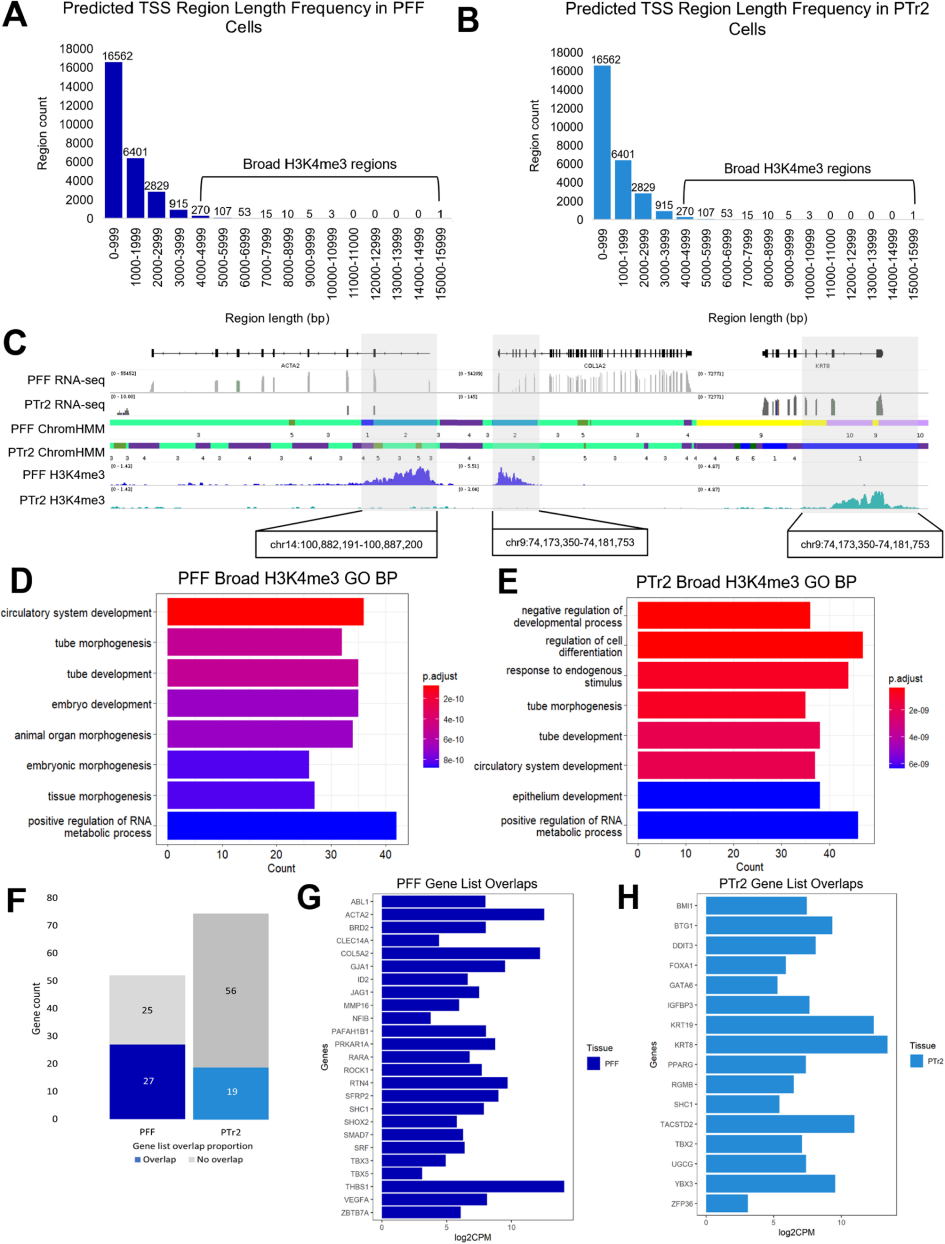
Characterization of broad H3K4me3 mark regions and associated genes in PFF and PTr2 cells. Histogram of lengths of global predicted TSS regions in (**A**) PFF and (**B**) PTr2 cells. (**C**) Genome browser view of broad H3K4me3 enrichment at three representative loci in both cell lines. Biological process (BP) annotation for broad H3K4me3 regions in (**D**) PFF and (**E**) PTr2 cells (*p* < O.05 using one-sided Fisher’s exact test; adjusted *P* < 0.05 after Benjamini-Hochberg FDR). (**F**) Proportions of genes found both in the list of genes associated with the top 5 overrepresented BPs for broad H3K4me3 regions and in the top 10% of genes identified as cell-type specific using transcriptional data. Identities of genes overlapping the two list categories in (**G**) PFF and (**H**) PTr2 cells

While the majority (around 87–88% in both cell lines) of regions in predicted TSS states (emission states 1 and 2) were 2 kb in length or less, a small subset of regions spanning 4 kb or more were identified in both PFF and PTr2 cells. In PFF cells, 464 regions were found to be spanning at least 4 kb in length, representing 1.70% of the putative TSS regions that were identified in this cell line (Fig. 4A), while in PTr2 cells, 593 regions spanning at least 4 kb were found, representing 1.80% of all predicted PTr2 TSS regions (Fig. 4B). These regions were strongly enriched in H3K4me3, and genome browser evaluation of their coordinates revealed a variety of genes marked with this broad H3K4me3 pattern (Fig. 4C). Biological functional annotation of these broad H3K4me3 regions in both cell lines revealed that they were strongly associated with genes involved in developmental processes (Fig. 4D, E; Supplementary Fig. S6A, B). Additionally, genes marked with broad H3K4me3 were expressed (Supplementary Fig. S6C, D). When these gene associations from the top five most overrepresented biological processes were compared to genes identified as either PFF or PTr2-specific (using a *t-*statistic ranked list approach on RNA-seq data), 52% of the genes in the PFF biological process list were present in the top 10% of PFF-specific genes (27/52), while this proportion was just over 25% in PTr2 cells (Fig. 4F). According to RNA-seq data, these genes were consistently transcriptionally active in both cell lines (Fig. 4G, H).

## Discussion

The functional organization of chromatin serves not only to efficiently package the genome within the nucleus but also to enable the modulation of gene expression across different developmental time points and in response to various stimuli. Early development in mammals involves the precise and dynamic regulation of gene expression by epigenetic mechanisms, an essential group of processes that govern differentiation, lineage specification, and the preservation of identity across multiple cell divisions. Epigenetic landscape studies during early development can help us better understand how gene expression is regulated across different developmental time points, and these details can also be used for comparison in studies focused on evaluating aberrant developmental circumstances. However, despite gradual progress by FAANG and similar initiatives to remedy the relative dearth of epigenetic information available for domestic species, many gaps remain in our knowledge surrounding the epigenetic landscape in these species, especially regarding histone marks and chromatin state during early development. Continued efforts in this area are especially important given the immense combined value of the pig as a biomedical model and as a staple food animal species. The results of this study provide detailed enrichment characterizations of H3K4me3, H3K27ac, H3K27me3, and BRG1 in PFF and PTr2 cells, two porcine lines with many established applications in the fields of reproductive and molecular biology. Additionally, we further demonstrate that chromatin state prediction maps can be used to identify important epigenetic regulatory regions potentially involved in governing cell identity.

Our analysis of H3K4me3 enrichment in PFF and PTr2 cells revealed that the vast majority of H3K4me3 sites in both cell lines were in promoter regions around transcriptional start sites of transcriptionally active genes, an observation that is consistent with the character of this mark as reported in the literature. H3K4me3 is one of the most widely profiled histone marks and is known to be enriched around active promoters during embryonic development and beyond [52–57]. Similarly, H3K27ac showed enrichment at promoter regions, though relatively less than H3K4me3, likely due to its presence at both promoters and enhancers [58, 59]. In both PFFs and PTr2s, several thousand (8,705 in PFFs and 7,416 in PTr2s) H3K4me3 and H3K27ac enrichment sites had overlapping genomic coordinates. The interplay between these PTMs has been investigated [60–62] and it has been posited that the upstream presence of H3K27ac may guide the installation of H3K4me3 at promoters by the acetylated histone reader BRD2 [62], though investigations into how H3K4me3 and H3K27ac may cooperate to influence transcription are ongoing.

H3K27me3, which is generally associated with transcriptional repression, showed lower enrichment at gene start and end sites compared to H3K4me3 and H3K27ac, mainly appearing in intergenic regions. Some active genes exhibited both H3K4me3 and H3K27me3 marks, possibly indicating bivalent regulation. The protein encoded by *FGF9*, the sole gene bound by both H3K4me3 and H3K27me3 in PFFs in the overrepresentation analysis, is known to have many roles in early development, including sex determination [63], cell proliferation [64], and morphogenesis [65–68]. Bivalent regulation of developmentally associated genes has been reported [69, 70], including *FGF9* [71–73], and while it is perhaps more commonly associated with pluripotency maintenance, it has been posited that this regulatory pattern may also play a role in modulating tissue-specific gene expression during later stages of development and into adulthood [69]. Indeed, in both cell lines, H3K27me3 was most enriched for developmentally associated terms relative to the other marks. Genome browser exploration of mark enrichment at these developmentally-related genes showed that some genes were bivalently marked with H3K4me3 and H3K27me3, while others were marked with only H3K27me3. RNA-seq data for these genes showed variable expression levels, and genes marked only with H3K27me3 were broadly not expressed according to this data. For example, *BMP4* was present in the list of developmentally-associated genes marked with H3K27me3 in PFF cells, but the corresponding RNA-seq data indicated that this gene was expressed, albeit at relatively low levels, in this cell type. *BMP4* is known to be implicated in a variety of developmental processes such as tissue morphogenesis [74–77]. Genome browser views of the *BMP4* locus indicate the enrichment of both H3K4me3 and H3K27me3, with H3K27me3 signal at and/or immediately upstream of gene promoter regions. In contrast, *KLK4*, an ameloblast-associated gene [78], was also in this list of H3K27me3-enriched genes, but it was not expressed based on RNA-seq data. Examination of the epigenetic status of this gene indicated that it was marked only by H3K27me3, consistent with its repressed character in this cell type.

BRG1 (for Brahma-related gene 1; encoded by SMARCA4) is one of two mutually exclusive central ATPases present within mammalian SWI/SNF (BAF) chromatin remodeling complexes, along with Brahma (BRM, encoded by SMARCA2). BRG1 contains a bromodomain that is capable of recognizing and binding acetylated histone lysine residues [79, 80], an event that results in SWI/SNF recruitment to genomic regions. Our analysis of global BRG1 enrichment in PFFs and PTr2s revealed that while some BRG1 presence could be detected at promoters, the vast majority of this remodeling enzyme’s signal was detected outside of promoter regions, specifically in introns and distal intergenic stretches. RNA-seq data indicated that BRG1 was present at or around some transcriptionally active genes marked with H3K4me3, but not always consistently. BRG1 localization to promoter regions has been shown to initiate transcription following SWI/SNF-dependent chromatin remodeling [81–83]. There is also evidence to suggest that BRG1 can function as a transcriptional silencer [84] or regulator of H3K4me3 levels at promoters [85], highlighting the capacity of the SWI/SNF chromatin remodeling complex to exert versatile transcriptional control. BRG1 can bind both promoters and enhancers, with aberrant BAF complex activity affecting enhancer function and bivalent promoter regulation in cancers [86–93]. Our results indicated that a large number of transcriptionally active genes involved in the regulation of cell population proliferation and organism development were enriched with both BRG1 and H3K27ac in PFFs, which may suggest that chromatin accessibility at these genes is partly regulated through a mechanism facilitated by the recognition of H3K27ac by the BRG1 subunit of SWI/SNF complexes, though further examination of this relationship with additional chromatin profiling techniques would be needed to provide any mechanistic conclusions. The lack of genes identified to be co-bound by BRG1 and H3K27ac in PTr2 cells could be due, in part, to the stringency of the applied statistical parameters, but it may also be at least partly reflective of a relatively lower frequency of BRG1/H3K27ac co-occupancy in this particular cell line.

The chromatin state prediction maps generated using these epigenetic features showed widespread conservation of certain regulatory regions such as TSSs between the two cell lines, except at cell type-specific genes. Additionally, transcription factor motif analysis of putative enhancer elements showed some similarities in motif conservation between the two cell lines, including the FOSL/JUND heterodimer, and TEAD family transcription factors, each of which have several known associations with regulating fundamental early developmental processes [94–97]. The transcription factor motifs that differed between PFF and PTr2 cells still included transcription factors with established implications in developmental processes such as cell proliferation, differentiation, and cell fate determination, suggesting that these differences may be involved in the coordination of cell type-specific gene expression programs. The chromatin state maps also depicted discernable differences in TSS/promoter region lengths and facilitated the identification of a subset of genomic regions marked with broad (> 4 kb) H3K4me3 signal. Most H3K4me3 enrichment is 1–2 kb in length immediately upstream and towards the 5’ end of gene bodies, though a subset of broad H3K4me3 enrichment patterns ranging from around 4 kb to upwards of 60 kb in length has been reported and associated with maintenance of cell identity [98–100]. Our findings in PFF and PTR2 cells support the observations that broad H4K3me3 domains may be implicated in regulating the expression of cell type-specific genes, as these subsets were strongly associated with genes involved in essential developmental processes, including transcriptionally active genes identified as being in the top 10% of cell type-specific genes in each cell line. Analysis of RNA-seq data indicated that genes marked by broad H3K4me3 in PFF and PTr2 cells are expressed, suggesting that broad H3K4me3 may have an activating role in these cell types. Broad H3K4me3 is reportedly associated with consistent and relatively high gene expression levels in many cell types [98, 101–103]. However, there is also evidence to suggest that broad H3K4me3 may have more repressive roles in certain contexts, such as in early embryo development, where broad H3K4me3 regions must be removed in order for zygotic genome activation (ZGA) to proceed [57, 104–106]. Given the need for precise temporal and spatial regulation of gene expression and the dynamic nature of chromatin organization during early embryonic development, the broad H3K4me3 domain may serve as an example of how epigenetic mechanisms exert their regulatory functions in a context-dependent manner.

In a 2021 paper, Pan and colleagues created chromatin state prediction maps across 14 different porcine tissues as part of a broader effort to construct the beginnings of an atlas of functional genetic elements in this species [21]. Part of this research was to bring attention to efforts by the Functional Annotation of Animal Genomes (FAANG) Consortium to bring together datasets produced by RNA-seq, chromatin profiling, DNA methylation studies, and chromatin interactions and accessibility in domestic livestock to address the relative lack of genomic annotation in these species [107]. While the chromatin state details from the present work are not as comprehensive and informative as the aforementioned study (which combined ChIP-seq, ATAC-seq, Reduced-representation bisulfite sequencing (RRBS-Seq), and RNA-seq), they do represent two cell types and developmental time points not presently captured in the atlas. Indeed, the authors of the Pan et al. study conclude that much greater quantities of epigenomic data will need to be collected across more developmental stages and tissues, particularly reproductive tissues, to develop a deeper understanding of the genotype-to-phenotype relationship and the complex underlying mechanisms and functionalities that influence development and disease.

## Conclusions

Taken together, these findings provide a view of the epigenetic landscape and predicted chromatin state in PFF and PTr2 cells, highlighting both the similarities and differences in epigenetic feature and regulatory region localization involved in maintaining essential functions and conferring unique cell identity. As pigs continue to grow in popularity as biomedical models for human-oriented research, likely, concerted efforts to understand the mechanistic underpinnings guiding gene expression in this species will continue to be examined and compared to human contexts in order to maximize the utility and efficiency of using swine as a model organism for translational research.

### Electronic supplementary material

Below is the link to the electronic supplementary material.

Supplementary Material 1

Supplementary Material 2

Supplementary Material 3

Supplementary Material 4

Supplementary Material 5

Supplementary Material 6

Supplementary Material 7

## Data Availability

The CUT&RUN datasets generated and analyzed during the current study are available in the NCBI Sequence Read Archive (SRA) under BioProject PRJNA1036691. The RNA-seq data analyzed during the current study are available for download from the NCBI SRA BioProjects PRJNA798047 and PRJNA778857.

## Abbreviations

BRG1: Brahma-related gene 1
CUT&RUN: Cleavage under targets & release using nuclease
EZH2: Enhancer of zeste homolog 2
FGFR2: Fibroblast growth factor receptor 2
FAANG: Functional Annotation of Animal Genomes
GO: Gene Ontology
KGFR: Keratinocyte growth factor receptor
NGS: Next-generation sequencing
pAG-MNase: Protein A/G micrococcal nuclease
PFF: Porcine fetal fibroblast
PTM: Post-translational modification
PTr2: Porcine trophectoderm
RRBS-seq: Reduced-representation bisulfite sequencing
SCNT: Somatic cell nuclear transfer
TES: Transcriptional end site
TSS: Transcriptional start site
ZGA: Zygotic genome activation
