# Supplementary material for: Chromatin profiling and state predictions reveal insights into epigenetic regulation during early porcine development
Source: Epigenetics Chromatin. 2024 May 21;17:16. doi: 10.1186/s13072-024-00542-w (PMC11106951; doi:10.1186/s13072-024-00542-w)
Supplement: Supplementary file 1 — Supplementary Material 1 [file 13072_2024_542_MOESM1_ESM.pdf]

Supplementary Figures

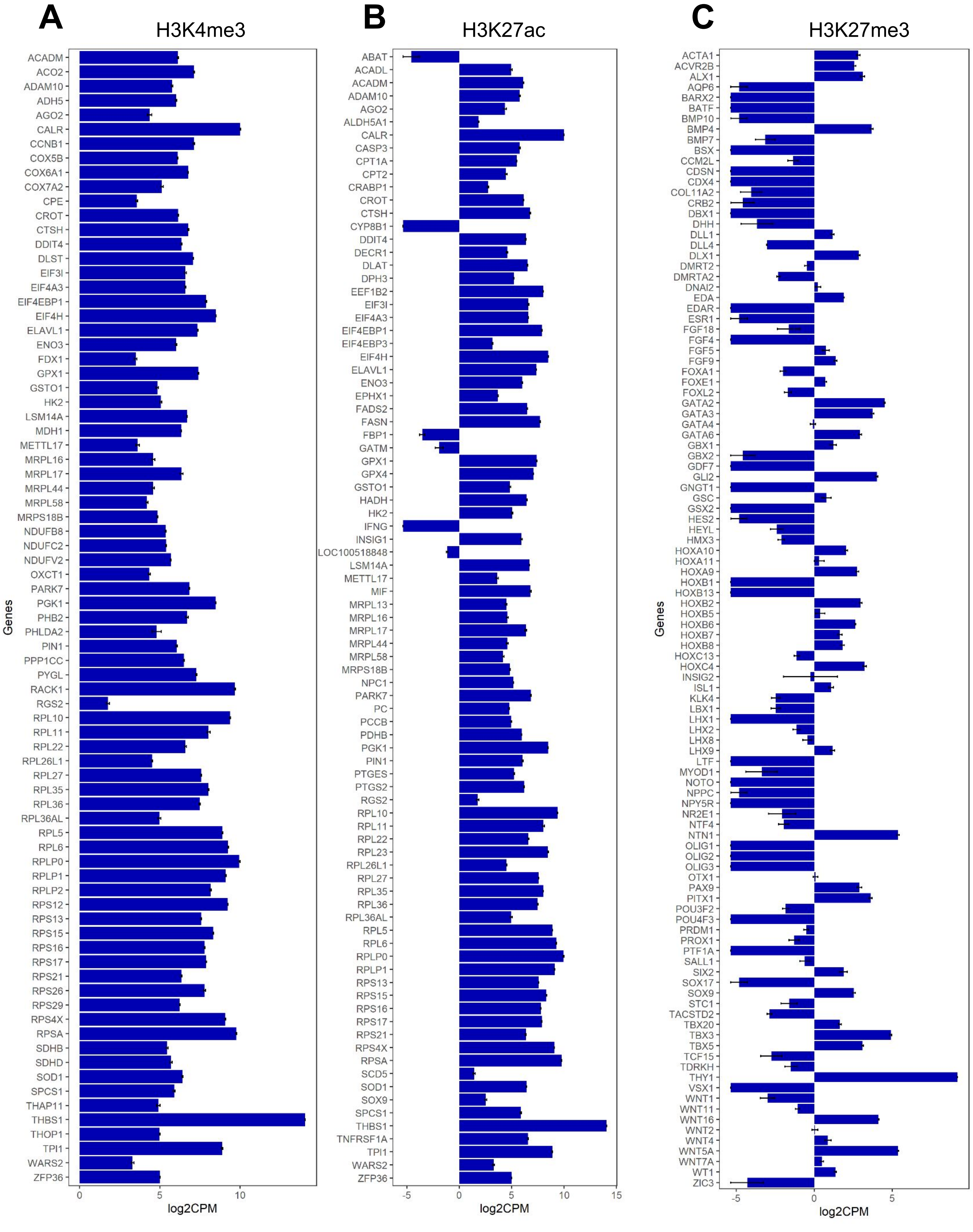

Supplementary Figure S1. Gene lists and associated expression values for genes in the top 3 most-enriched GO biological processes for (A) H3K4me3, (B) H3K27ac, and (C) H3K27me3 in PFF cells. Expression values are given as mean log2CPM, and error bars indicate standard error.

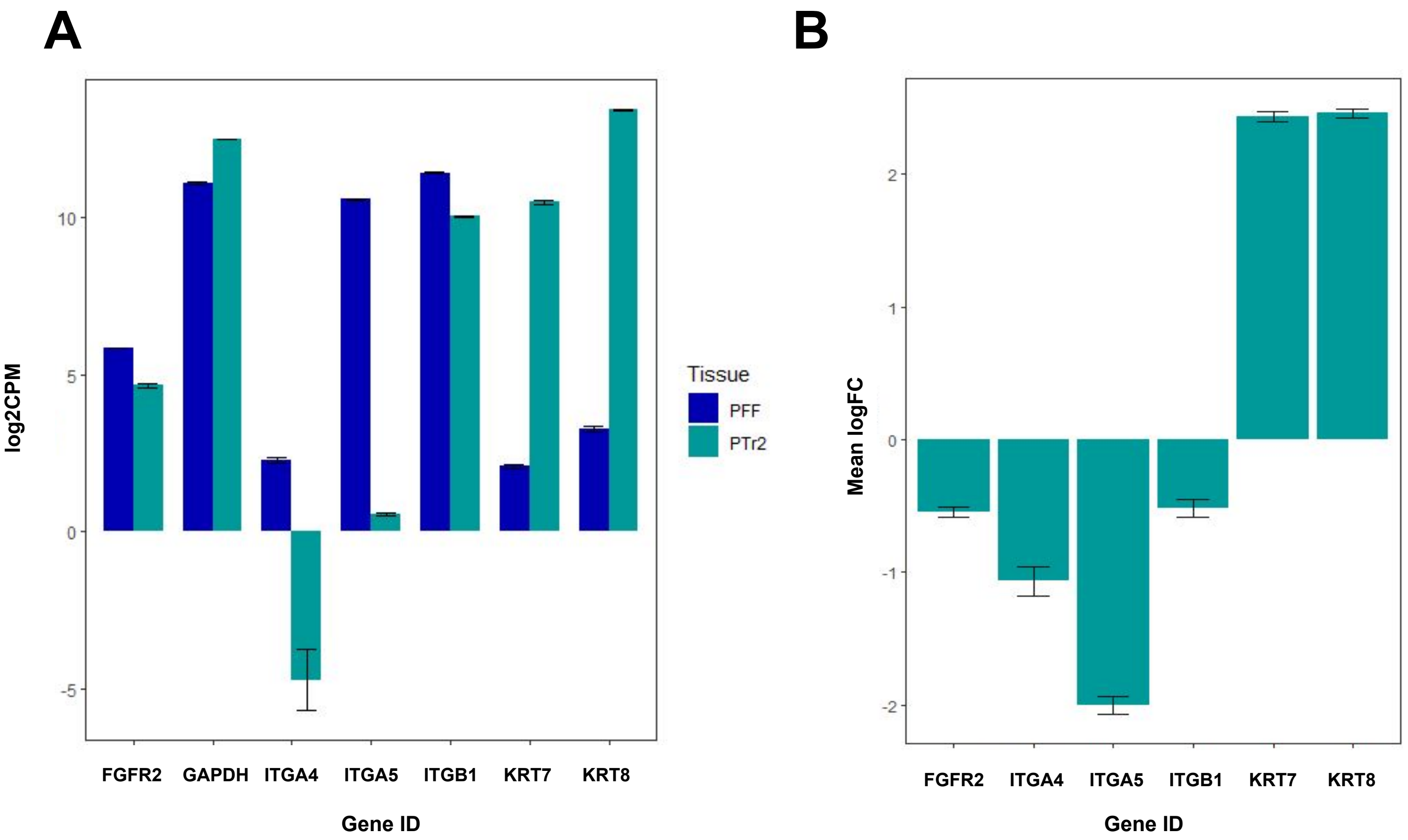

Supplementary Figure S2. Gene expression of the genes selected to characterize PTr2 cells. Gene expression was evaluated via (A) publicly-available RNA-seq data and (B) RT-qPCR. For the RNA-seq data, expression values are given as mean log2CPM. GAPDH was included to confirm stable expression in both cell lines prior to its use as a housekeeping gene. For the qPCR data, fold changes were calculated in PTr2 cells relative to PFF cells and log transformed. Error bars denote standard error.

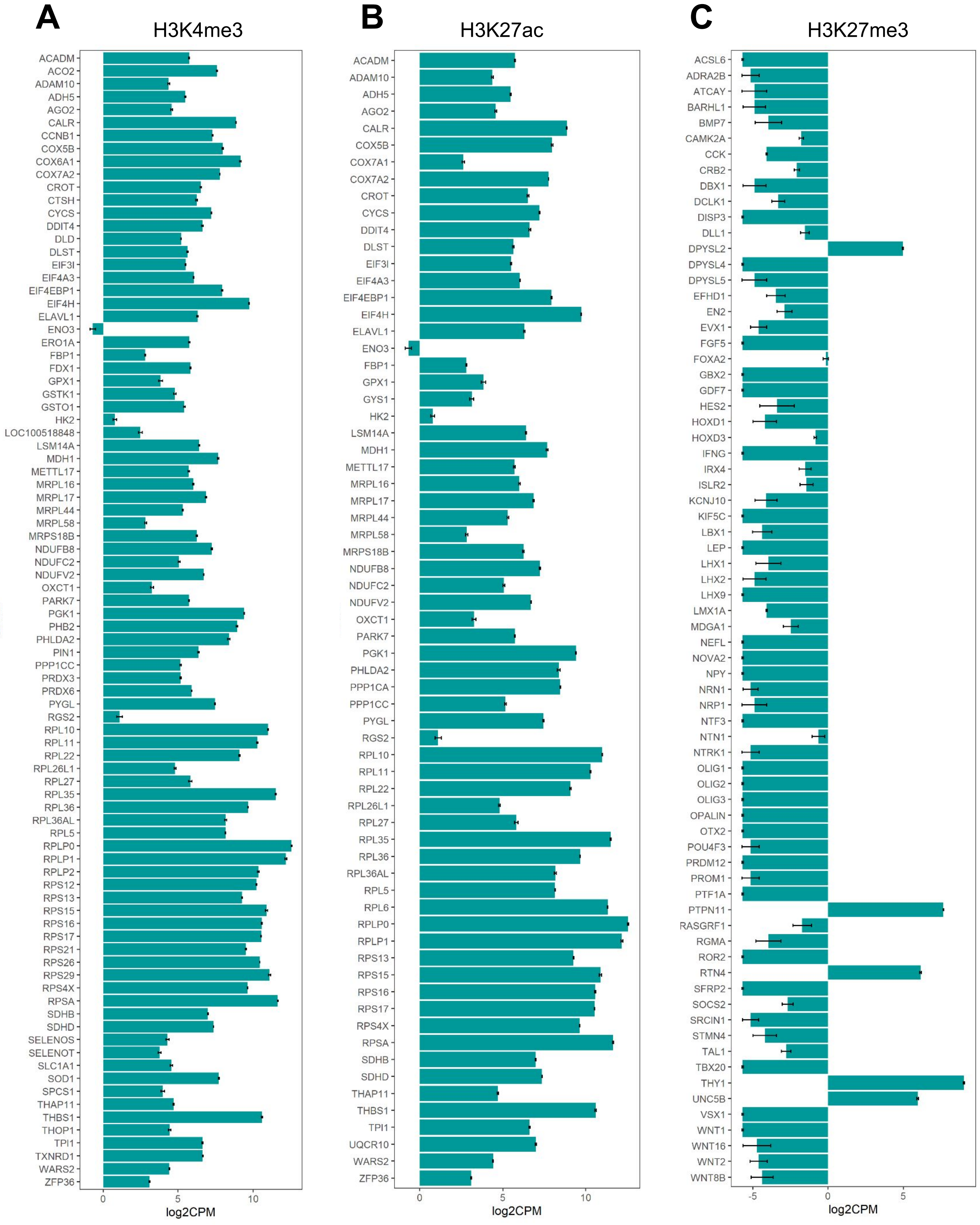

Supplementary Figure S3. Gene lists and associated expression values for genes in the top 3 most-enriched GO biological processes for (A) H3K4me3, (B) H3K27ac, and (C) H3K27me3 in PTr2 cells. Expression values are given as mean log2CPM, and error bars indicate standard error.

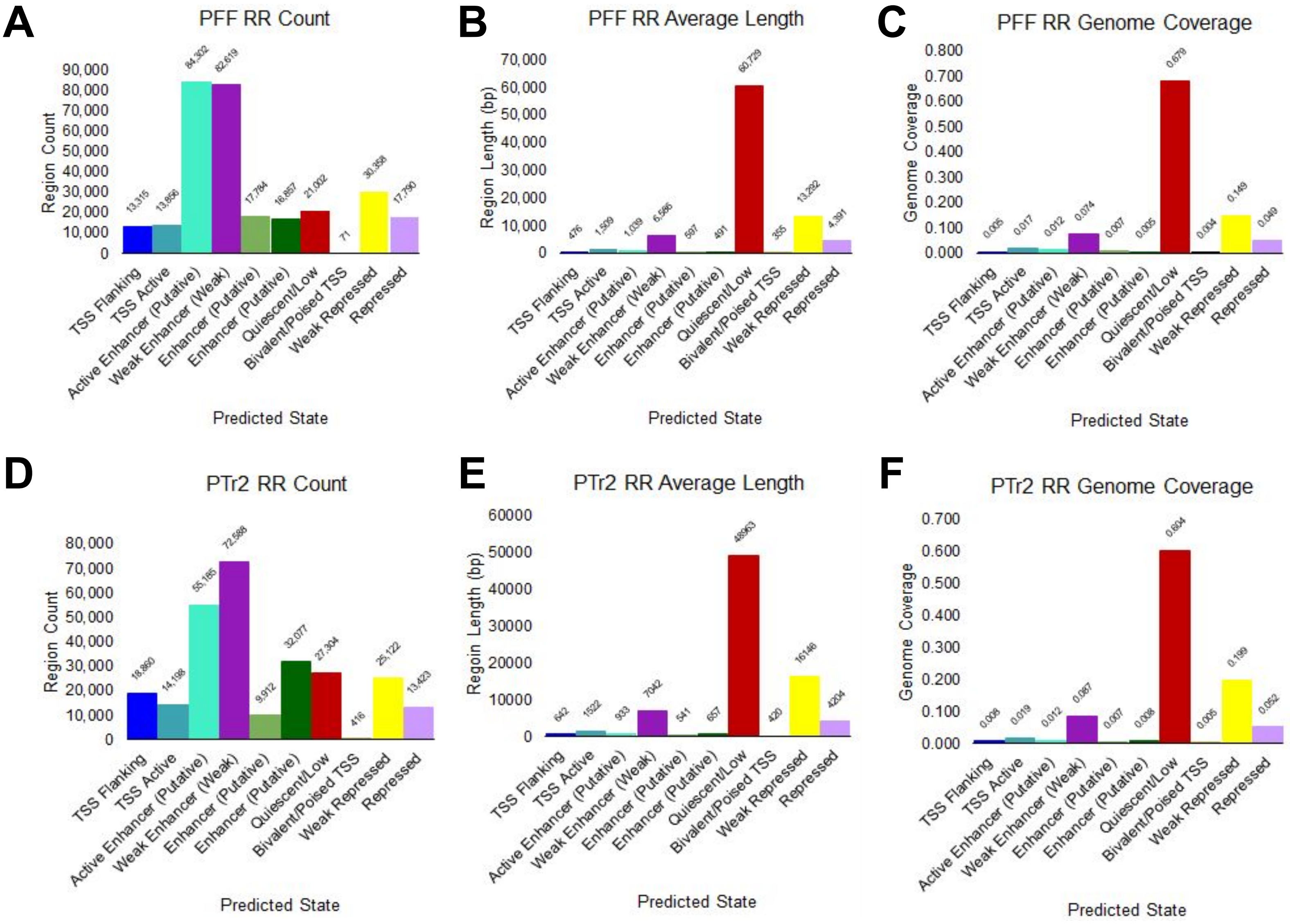

Supplementary Figure S4. Global counts, average lengths, and relative coverage of each predicted regulatory region (RR; equivalent to chromatin state) in (A-C) PFF and (D-F) PTr2 cells.

		Enriched motif	Motif identity	% Targets with motif	p-value
PFF	1.	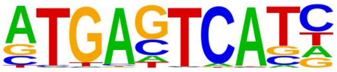	FOSL1::JUND	6.84	1e-939
	2.	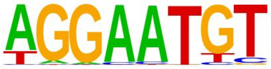	TEAD2	12.06	1e-158
	3.	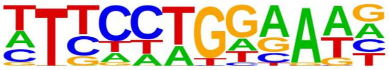	BCL6	29.01	1e-115
	4.	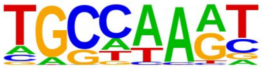	NFIA	28.77	1e-109
	5.	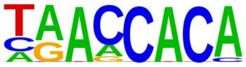	RUNX	18.75	1e-105
PTr2	1.	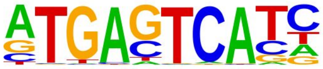	FOSL:1:JUND	8.26	1e-1453
	2.	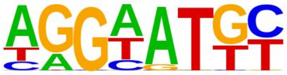	TEAD4	22.68	1e-281
	3.	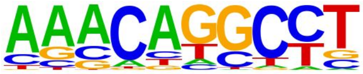	ZFX	35.63	1e-159
	4.	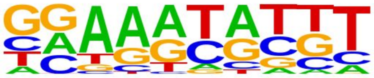	CCA1	36.79	1e-153
	5.	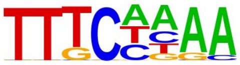	CEBPD	46.83	1e-135

Supplementary Figure S5. Transcription factor motif enrichment analysis for putative enhancer regions. For each cell type, the top 5 (by statistical significance of enrichment) enriched motif sequences are included, along with their corresponding identities, the percentage of input regions containing each motif, as well as the enrichment p-values for each motif.

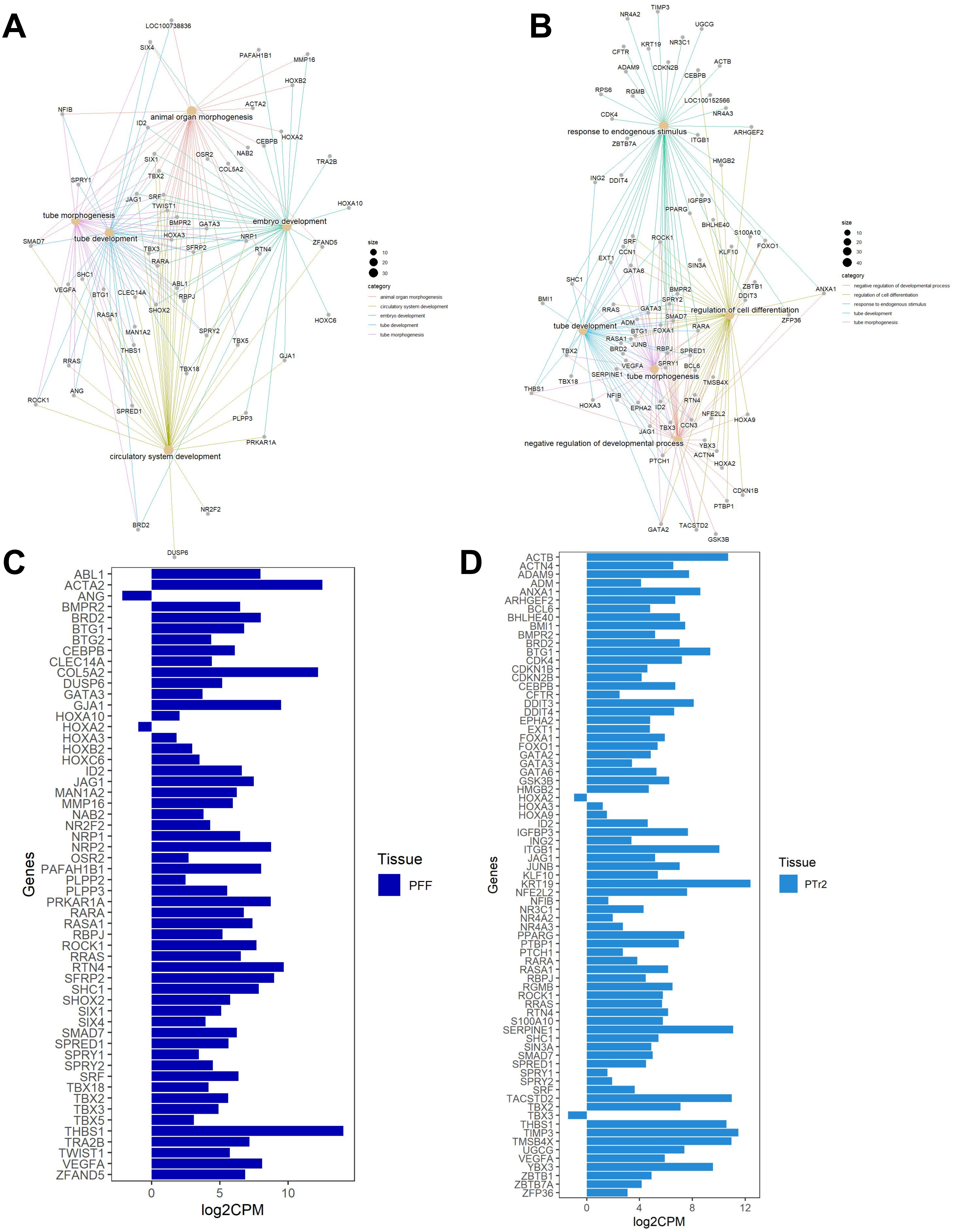

Supplementary Figure S6. Gene networks for the top 5 biological processes associated with broad H3K4me3-bound regions in (A) PFF and (B) PTr2 cells. (C) RNA-seq data (log2CPM) is provided for all genes in these categories for (C) PFF and (D) PTr2 cells.
